# Inference on COVID-19 Epidemiological Parameters Using Bayesian Survival Analysis

**DOI:** 10.3390/e23101262

**Published:** 2021-09-28

**Authors:** Chiara Bardelli

**Affiliations:** 1 Department of Mathematics, University of Pavia, 27100 Pavia, Italy; chiara.bardelli01@universitadipavia.it; 2 Department of Political and Social Sciences, University of Pavia, 27100 Pavia, Italy

**Keywords:** forecasting, COVID-19, uncertainty quantification, Bayesian Survival Analysis

## Abstract

The need to provide accurate predictions in the evolution of the COVID-19 epidemic has motivated the development of different epidemiological models. These models require a careful calibration of their parameters to capture the dynamics of the phenomena and the uncertainty in the data. This work analyzes different parameters related to the personal evolution of COVID-19 (i.e., time of recovery, length of stay in hospital and delay in hospitalization). A Bayesian Survival Analysis is performed considering the age factor and period of the epidemic as fixed predictors to understand how these features influence the evolution of the epidemic. These results can be easily included in the epidemiological SIR model to make prediction results more stable.

## 1. Introduction

The World Health Organization (WHO) declared a pandemic on 11 March 2020, for the outbreak of Coronavirus Disease 2019 (COVID-19), caused by Severe Acute Respiratory Syndrome Coronavirus 2 (SARS-CoV-2) [1]. Since the start of the pandemic, the novel COVID-19 spread all across the world, infecting more than 130 million individuals and causing over 2.8 million deaths by 4 April 2021 [2]. These huge numbers show a critical emergency situation which involves all the six macro areas identified by the WHO. After more than a year of the pandemic, national health care systems and, as a consequence, global and local economies are still under pressure, especially in the Americas, Europe and India. Globally, nationally, and at every sub-governmental level, the need to monitor the ongoing spread of the disease has become more and more fundamental to guide public health awareness and political responses [3]. One of the most important tools to address the monitoring problem is the development of mathematical and statistical models based on past available data. These models can predict the evolution of the phenomenon giving the opportunity for global and local institutions to coordinate the health care workforce and resources in an efficient way and to make informed decisions.

To provide an accurate prediction of the evolution of the epidemic, it is necessary to estimate the epidemiological parameters which best characterize the spread of the disease. It is important to take into consideration the uncertainty of these parameters and the temporal evolution of the quantities under consideration to implement successful strategies for the containment of the disease: on one hand the estimation of the transmission rate is a key point to realize successful social distancing measures and lockdown policies; on the other hand, analyses on the total length of hospital stay and the recovery times are crucial for optimal planning in hospitals to satisfy the bed demand.

From the point of view of the transmissibility, the presence of different variants of the COVID-19 virus all over the world [4] makes the study of the dynamics of the disease more complicated. Furthermore, the effects of lockdown strategies cannot be measured instantaneously but results show an intrinsic delay which makes the outcome of interventions difficult to assess and to predict [5,6]. On the other hand, different studies have been conducted for analysis of length of hospital stay and, more in general, on the recovery times which characterize the COVID-19 disease [7,8]. The estimation of the total length of stay in hospital combined to the prediction of hospital admission rates allows quantification of the bed demand and to organize the hospital activities best [9]. This challenging task requires a careful modelling approach since high quality data are often limited to fragmented data sources from different hospitals.

Furthermore, the estimation of recovery times of COVID-19 is also useful to address the nonidentifiability problem [10]: in the SIR model [11] and all its extensions (SEIR [12], SIRD [13], etc.) many different combinations of epidemiological parameters can show a good fit to real data during the calibration phase, but they can produce very different predictions of the epidemic. This greatly influences the reliability of model predictions and makes the choice of epidemiological parameters more challenging. In [14] different epidemiological models are compared to study the accuracy of the predictions with respect to confirmed cases. Simple models, like the SIR one which defines only three sections of the population (Susceptible, Infected and Recovered), seem to perform better than more complex models despite the mild assumptions. The nonidentifiability problem in model calibrations is the main reason for such wide differences in predictions provided by the models. This problem has been addressed in [15] where, thanks to a preliminary statistical analysis based on historical data, parameters of the epidemiological Social SIR model are best fitted.

The aim of this work is to analyze epidemiological parameters related to times which characterize the natural history of COVID-19 disease: Time to Viral Clearence (TVC), Delay in the Hospitalization (DH) and Length of Stay in hospitals (LoS). The estimation of all these quantities can be helpful for the calibration of the SIR model and its extensions. TVC can approximate the inverse of the recovery rate (γ) in a simple SIR model, while LoS and DH are useful for epidemiological models which divide the population in a higher number of compartments (for example the compartment of hospitalized people) like in [16,17].

We develop a statistical model able to consider both, the uncertainty of data at hand, and the results found in the literature about the quantity of interests, applying a Bayesian approach. The statistical model will be tested on a dataset restricted to the area of Pavia, a province of Lombardia, in north of Italy. The dataset is provided by Health Protection Agency (ATS) of Pavia and includes all the confirmed COVID-19 cases of the province and the patients hospital stays in days. Despite data being limited to a small Italian geographic area, they refer to a population of more than 500,000 people.

The paper is organized as follows: the first part of Section 2 describes the dataset provided by ATS of Pavia made up of interesting insights about the historical evolution on COVID-19, while in the second part the Bayesian Survival model is presented; in Section 3, the results of the approach applied to data are discussed; Section 4 shows an application to the Social SIR model. Section 5 reports some conclusions and remarks.

## 2. Material and Methods

### 2.1. The Study Population

The statistical analyses were performed on the dataset provided by the ATS of Pavia (Italy), which includes all the confirmed cases of COVID-19 registered from 21 February 2020 to 17 January 2021, and covers a wide period characterized by different behaviors in the spreading of the disease. It represents a local context in terms of geographical area (only 3000 km^2^, considering the 300,000 km^2^ of the entire Italian territory). However, the susceptible population of this area is about half a million of people, a sufficiently large number for obtaining statistically significant results. Furthermore, this empirical analysis can prove how data can be used to describe the evolution of the epidemic in a restricted area and support decisions of local institutions which can be easily extended to national level.

For each COVID-19 patient of the dataset, all the relevant dates which describe the personal evolution of the disease are reported, in particular: date of the first positive swab test, date of the first negative swab test which is considered the recovery date, death date, admission and discharge date in cases of hospitalization. Age and sex of each COVID-19 patient recorded in the dataset are also available. Thanks to this detailed information, it is possible to statistically estimate the epidemiological parameters of the disease leading to a more realistic prediction of the spread of COVID-19 infection in the province of Pavia. The accuracy of these results, instead, can not be achieved when analyzing open datasets (like [18,19]), which often provide only the aggregated quantities of different compartments of the population.

**Figure 1:**
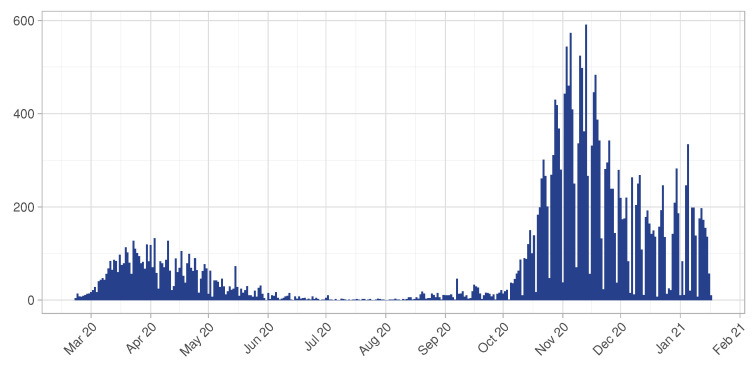
Confirmed COVID-19 cases in the province of Pavia at 18 January 2021.

In Figure 1 the number of confirmed COVID-19 cases per day is displayed, for a total of 26,763 infectious individuals and 2014 cumulative deaths during the considered period. Notice that the province of Pavia has been interested from two different peaks, in line with the general trend of the disease spread recognized in the North of Italy during the same period. The second peak is still ongoing, showing a pretty stable situation in terms of number of new cases per day.

**Figure 2:**
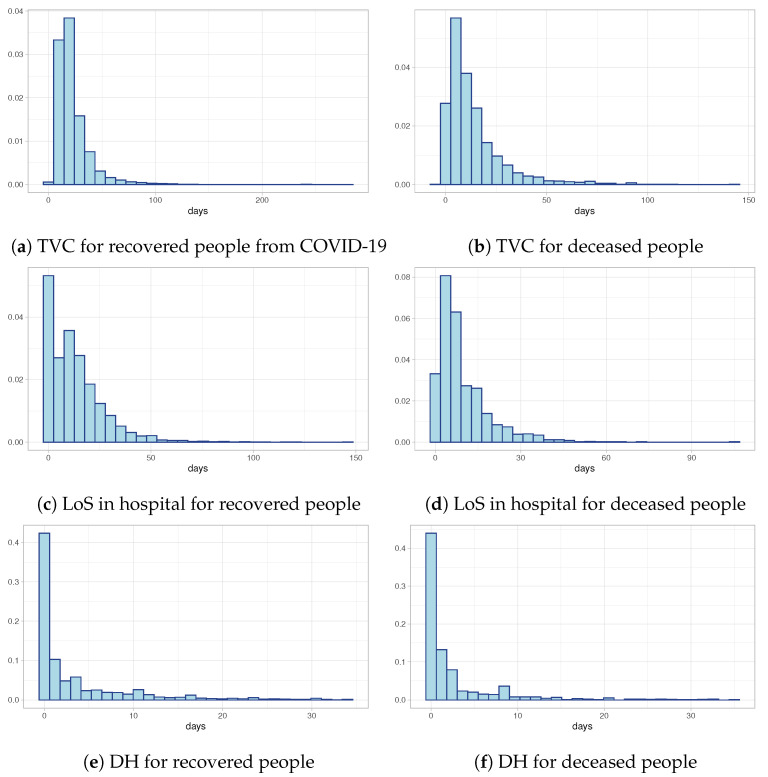
Histograms of TVC, LoS and DH for recovered and deceased people.

Figure 2a,b show the distributions of Time to Viral Clearance (TVC) computed as the difference between the first swab test (date of diagnosis) and the recovery date (in the case of recovery) or the death date (in the case of deceased individuals). Both of them show a strongly positive skewness with heavy tails. Despite the similar shape of the two distributions, the median value in the case of recovered individuals is equal to 19 days compared to 9 days for deceased individuals. These preliminary results are confirmed also by the distributions of Length of Stay in hospital (LoS) depicted in Figure 2c and Figure 2d, respectively, for recovered people and deceased people: the LoS in hospital for recovered people is longer than for deceased individuals. Finally, Figure 2e,f show the observed distributions of times between the first swab test and the hospitalization day for both recovered and deceased people. The histograms suggest that the majority of hospitalized people discover they are infected the same day they are hospitalized. All these distributions need a deeper study, both to understand if external predictors (like age or period of the epidemic) can influence positively or negatively the distributions, and to model censored data which are excluded from the exploratory analyses.

**Figure 3:**
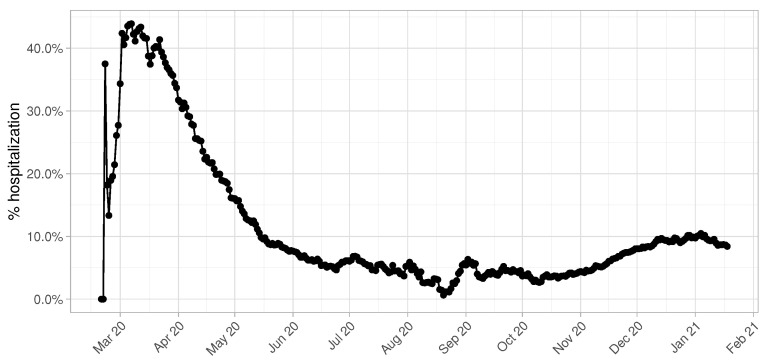
Percentage of hospitalized COVID-19 patients per each day.

Another fundamental parameter for accurate predictions in terms of number of beds demand is the percentage of hospitalized COVID-19 patients over the total number of infectious individuals. This value is highly influenced by the daily testing capacity of the province of Pavia, as we can clearly observe in Figure 3. During the first months of the epidemic, especially March and April, this value shows a peak achieving 50% and becomes more and more constant starting from the beginning of June. It is reasonable to consider the temporal window from June 2020 to January 2021 to give an accurate estimate of the real hospitalization rate, since data collected from March to May are altered by the low number of swab tests performed.

#### 2.1.1. Influence Analysis by Age Factor

**Figure 4:**
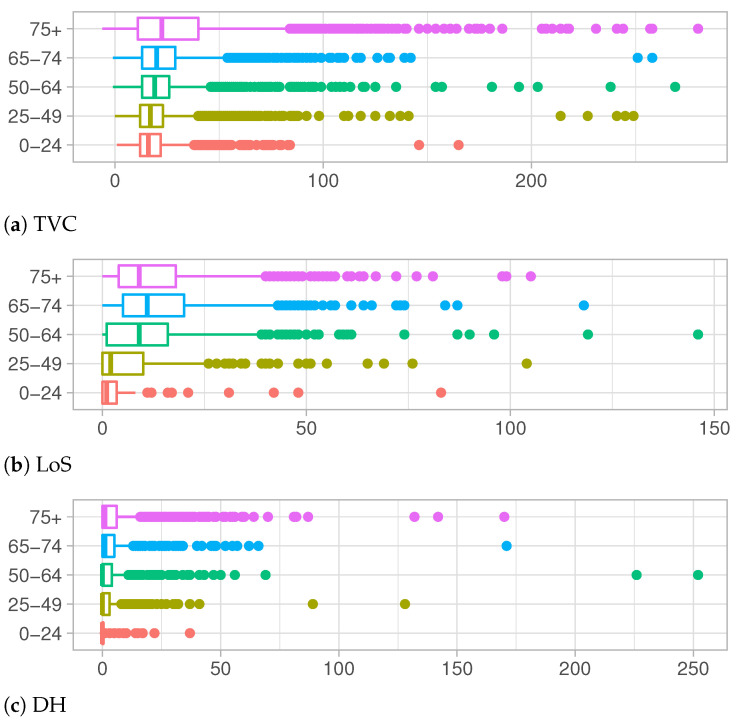
Distributions of TVC, LoS and DH for each age group.

Age is known to be a significant factor in the differentiation of personal evolution of the COVID-19 disease, in particular for the serious complications which can derive from the disease, are clearly more frequent in older people [20]. This evidence is confirmed by the data at hand represented in Figure 4a–c. They describe the observed distributions of Time to Viral Clearance, Length of Stay in Hospital and Delay in the Hospitalization, respectively, for each age group.

TVC values increase with the increase in age, both for median values and the third quartile of the observed distributions, as shown in Table 1a. Notice that even the support of distributions becomes wider and wider, showing a high number of outliers for the older groups. This behavior denotes a long recovery time for older adults, which are at higher risk for severe illness from COVID-19 [21]. In Figure 4b and Table 1b, a different trend is depicted. The Length of Stay in hospital increases for older age groups except for the over 75 years old category whose distribution presents shorter hospitalization times. This change in distributions can be explained keeping in mind that LoS is defined as the period between the admission in hospital and the discharge date (when patient recovers) or death date in case of deceased people. Adults in older age groups which are hospitalized have the greater risk of death and their health conditions can worsen very quickly.

The exploratory analysis on DH show similar shapes of distributions among different age groups in Figure 4c and Table 1c. Except for a few outliers, support of these distributions is restricted to lower values with respect to another quantity of interests. In particular, for younger categories, most people are tested the same day of the hospitalization or a few days later, meaning that, very often, the disease is not recognized until the day of hospitalization.

#### 2.1.2. Influence Analysis by Period of Epidemic

SARS-CoV-2 has evolved since it first emerged in late 2019. Several mutations of the virus have been observed: some variants appear more infectious than the original one; other variants, on the other hand, are characterized by a longer infectious period. As a consequence, the need to consider epidemiological parameters as dynamic quantities is becoming more and more evident.

In our work we define three periods of analysis characterized by different evolution of the epidemic and different containment measures applied in Italy. Period 1, from 21 February 2020 to 30 April 2020 is considered the first wave of the epidemic in Italy and overlaps with strict lockdown measures. Period 2 starts with 1 May 2020 and ends with 30 September 2020. It corresponds to the Italian summer, is characterized by a relaxation in lockdown measures and a significant decrease in the number of infected people. Contact tracing worked at best and hospitals could resume routine activities. Finally, Period 3 starting with 1 October 2020 and ending with 17 January 2021, is the second wave of the epidemic in Italy.

**Figure 5:**
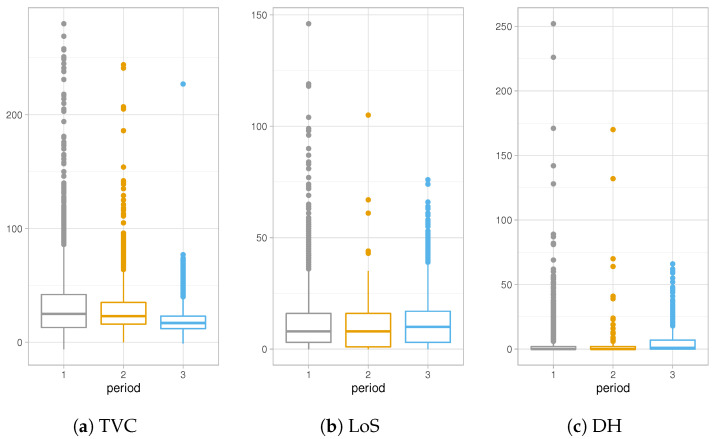
Distributions of TVC, LoS and DH in different periods of the epidemic.

Figure 5a–c show different trends in the three distinct periods of the epidemic. TVC distribution is characterized by greater values in Period 1 compared to the other Periods. This difference can be explained considering that collections of data during Period 1 have been affected by lot of imprecisions due to the overwhelming activities which have found the Italian healthcare system unprepared at the beginning of the epidemic. On the other hand, values of TVC show a decreasing trend, as shown also in Table 2a since symptoms of COVID-19 have been studied a lot and more efficient medical treatments have been used to contain the disease.

The results of Table 2b about LoS in hospitals show a slight decrease in the hospitalization in Period 2, apparently due to the fact that during summer months the epidemic was under control and hospitals were not under high pressure. Finally, Figure 5c and Table 2c present higher values for distribution of DH of Period 3, concluding that, recently, COVID-19 infectious people are recognized days before the hospitalization day.

### 2.2. Statistical Analysis

To estimate TVC, LoS and DH distributions, a Bayesian Survival Analysis was implemented considering age groups and the period of the epidemic as fixed predictors. The flexibility of the Bayesian statistical model allows one to make probability statements about parameters and to quantify uncertainties more easily. The possibility to integrate results from other research studies on the quantities of interest using prior functions is another advantage of the Bayesian framework. The combination of previous knowledge and data at hand allows one to better estimate the “true” underlying distributions of TVC, LoS and DH.

From a survival point of view, we assume that, for individual *i* (i=1,…,N), the baseline of the survival period (or observed entry time) and the event of interest are denoted $T_{i}^{E}$ and $T_{i}^{*}$, respectively, and their value change based on the type of the analysis:

- TVC $T_{i}^{E}$ is defined as the date of the first positive swab test; TVC $T_{i}^{*}$ is the recovery date for recovered people or death date for deceased people;
- LoS $T_{i}^{E}$ is defined as the hospitalization day; LoS $T_{i}^{*}$ is the discharge date for recovered people or death date for deceased people;
- DH $T_{i}^{E}$ is the first positive swab test and DH $T_{i}^{*}$ is the hospitalization day.

Patients who were still hospitalized on 17 January 2021 were included in the LoS analysis as correctly censored observations as well as people who were not recovered yet from the disease were considered correctly censored observations in TVC analysis.

For each type of analysis (TVC, LoS and DH), under a hazard scale formulation, different distributions were compared to model the hazard function which is the instantaneous rate that the event of interest happens. We include in this formulation the time-fixed linear predictors, age and period of the epidemic to understand if there are statistically significant differences between groups, as suggested by the exploratory analysis of Section 2.1. The final hazard functions are described by the following equation

(1) $$h_{i}\left(t\right)=h_{0}\left(t\right)exp\left(\eta_{i}\right)$$

where $h_{0}\left(t\right)$ is the baseline hazard and $\eta_{i}$ is the linear predictor.

The distributions of the three quantities of interest are approximated using different parametric models for the baseline hazards: the exponential model (EXP), the Weibull model (WEIB) and the cubic splines model with two and five internal knots. The Bayesian Leave-one-out cross-validation (LOO) was used to compare performances of the models.

For each of the parameters (baseline hazard function and linear predictors), a noninformative prior distribution is set. The choice of noninformative priors is motivated by the fact that the dataset under consideration is sufficiently rich to give robust estimates of the quantities of interest. On the other hand, previous studies (like [7,8]) regard only a restricted population of other European countries in a specific period of the epidemic, which usually show different trends with respect to the Italian epidemic. The parameters for the noninformative priors are the following: the regression coefficients are treated as a priori independent with normal priors centered at 0 and with standard deviation of 2.5; a half-normal distribution with location 0 and scale 2 is set for the Weibull shape parameter; a Dirichlet distribution parametrized by an all-ones vector is used for the auxiliary coefficients of the cubic splines model. To estimate the posterior distributions, the No-U-Turn Sampler [22], an implementation of Hamiltonian Monte Carlo, was used.

## 3. Results and Discussion

Considering the age factor or period as fixed predictors, the computation of LOO suggests that models with a flexible parametric baseline hazard (spline-based) fit the data best followed by the standard parametric models (Weibull, exponential), both in the case of TVC and LoS analysis. The analysis on DH suggests Weibull distribution as the best approximation for the hazard rate function. The following results refer to the models which provide the best approximations in terms of LOO.

### 3.1. TVC Results

**Figure 6:**
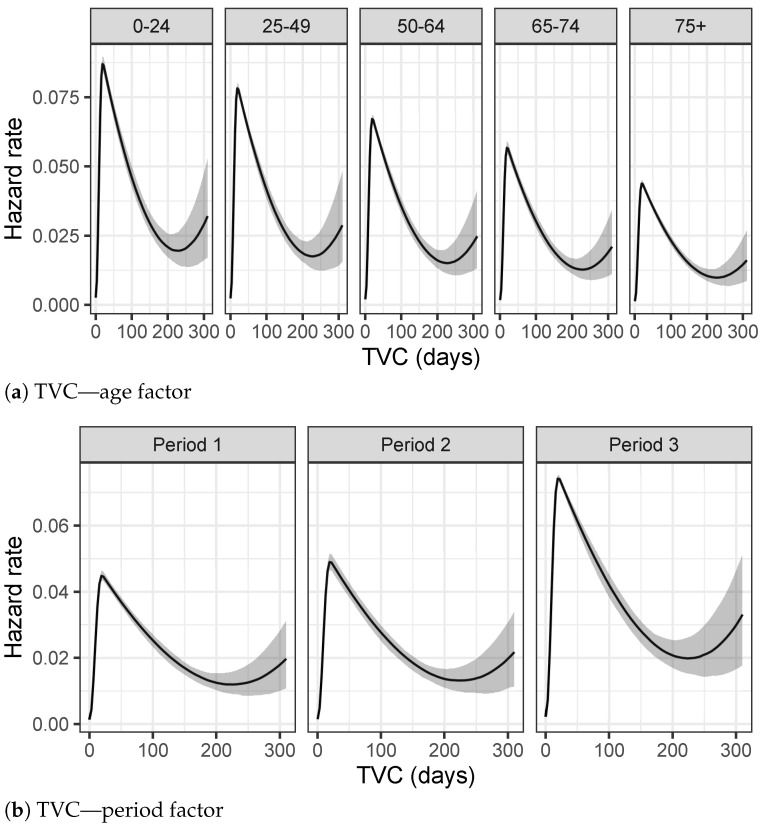
Hazard rates estimated using the splines model for each group.

The hazard rate, defined as the event rate at time t, differs a lot by age and period of the epidemic for TVC analyses. The highest peak of the hazard rate function, for people between 0 and 24 years old, is achieved at 16 days, as depicted in Figure 6a. This peak moves slightly toward larger values for older age groups, where it is less evident and the hazard rate function is more smooth, meaning that the youngest people have a higher probability of recovering in few days than older people. This behavior is also confirmed by Table 3a, where the Bayesian 95% credible intervals of the posterior TVC mean are listed for each age group. Considering period as a fixed predictor, both Figure 6b and Table 3b point out a significant reduction in terms of recovery times, including in the analysis also the right censored data, i.e., people who are still infectious on 17 January 2021.

### 3.2. LoS Results

**Figure 7:**
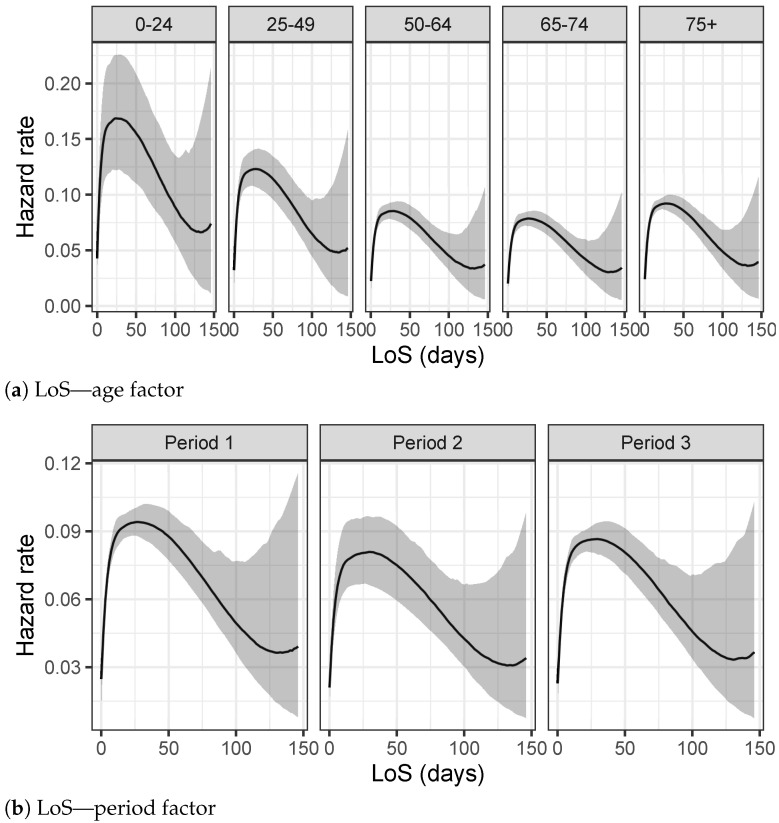
Hazard rates estimated using splines model for each group.

The analysis on LoS times in Figure 7a shows that the support of the hazard rate is restricted to smaller values with respect the analysis on TVC times. Notice that the shape of the hazard rate function of the over 75 group is in contrast with the trend of previous TVC analysis. LoS times show a peak for lower values meaning that the probability of being discharged from the hospitals for older people is higher in a range of LoS with low values. As already explained in the exploratory analysis of Section 2.1, this behavior is probably due to the fact that the analyses performed do not discriminate against recovered people from deceased people: the health conditions of the older people worsen very fast in the most complicated cases even if they are hospitalized, as also confirmed by Table 4a. As regards the period of the epidemic, the hazard rate function of Period 2 in Figure 7b shows lower values for the long time of hospitalization with respect to the other two periods under consideration. Table 4b confirms this trend with a 95% smaller credible interval for the mean of LoS.

### 3.3. DH Results

**Figure 8:**
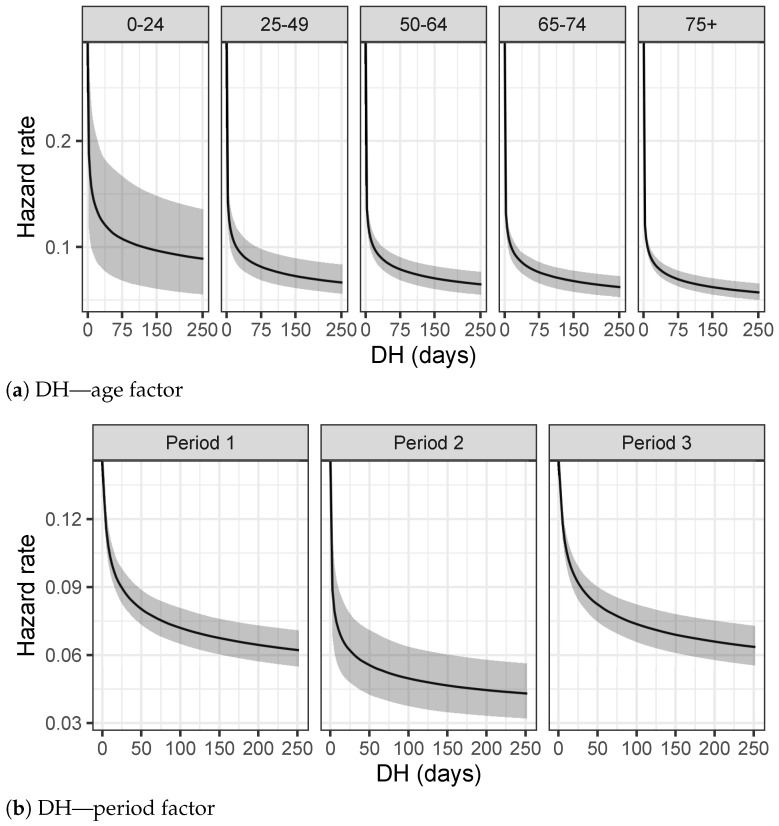
Hazard rates estimated using the splines model for each group.

Figure 8a on the Delay in Hospitalization shows a different behavior for the first age group, in which the shape of the hazard rate function is more uniform than the other ones. This result suggests that younger people are discovered to be infectious many days before they are hospitalized. Health conditions of younger people worsen more slowly than older people, since most of them do not have chronic conditions and are able to defeat the virus without any hospitalization.

Table 5a, as further evidence, presents similar results with the lowest values for the 95% credible interval of the 0–24 age group. On the other hand, Figure 8b reveals a strong difference in the Hospitalization Delay between the three periods. In particular, the hazard rate function of period 3 is more uniform, suggesting that infectious people are better recognized in an early stage of the disease and not the same day of the hospitalization, as confirmed also by the credible intervals of Table 5b.

## 4. Application to Social SIR Model

The results of the previous Section can be exploited to calibrate at best an epidemiological model. Here, a Social SIR model [15] is used to fit data provided by ATS of Pavia, following the equations:

(2) $$\frac{\partial S(t)}{\partial t}=-\bar{\beta }\;S\left(t\right)\;I\left(t\right)\;H^{2}\left(I\left(t\right)\right),$$

(3) $$\frac{\partial I(t)}{\partial t}=\bar{\beta }\;S\left(t\right)\;I\left(t\right)\;H^{2}\left(I\left(t\right)\right)-\gamma I\left(t\right),$$

(4) $$\frac{\partial R(t)}{\partial t}=\gamma I\left(t\right).$$

where $\bar{\beta }$ is the transmission rate, *H* is the function which models the social contacts and γ is the recovery rate.

**Figure 9:**
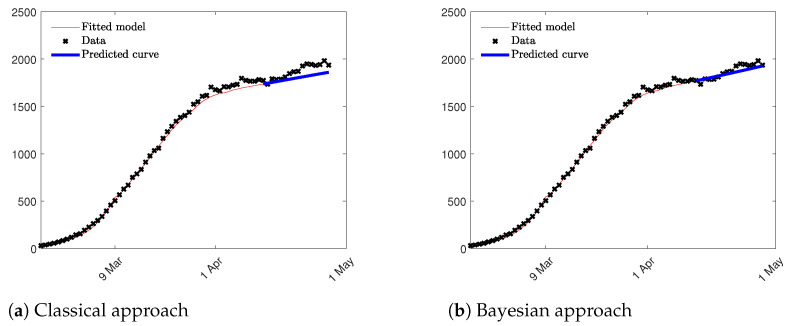
Epidemic curves estimated through the Social SIR model.

Two different approaches were compared: first, a Social SIR model where the parameters β and γ and the function *H* are estimated through an optimization process using the least squares method based on aggregated data of Susceptible, Infectious and Recovered; in the second approach, the γ parameter was estimated through a Bayesian Survival Analysis using the inverse of the mean of TVC given by its posterior distribution. This value was included in the Social SIR model as a fixed constant, and β and the *H* function were computed solving the traditional least squares problem. Since, in the second approach, the least squares method computes only two parameters; the impact of nonidentifiability problem is reduced and the estimation are more accurate as we can see in Figure 9. Notice that we compute both, the fitted model in red until 15 April, and the prediction epidemic curve in blue for the following two weeks. Root Mean Squared Error (RMSE) is used to validate the prediction results in the period of two weeks considered. RMSE is equal to 80.15 and 39.24, respectively, for the first and the second approach suggesting that the model which takes into consideration the results of the Bayesian Analysis fits better data at hand and, as a consequence, provides a better prediction result in term of the epidemic curve. In Figure 9 the two epidemic curves are represented confirming the results obtained through the RMSE index.

## 5. Conclusions

Accurate estimation of epidemiological parameters which describe COVID-19 epidemic is fundamental to develop robust predictive model whose aim is to forecast the evolution of the epidemic and help to realize successful social distancing measures and lockdown policies. The fragmentation of data and their imprecision must be taken into consideration when statistical and mathematical models are applied. Bayesian framework can help to handle the uncertainty of data and integrate previous results in the analysis of observed datasets. In this paper, we model different quantities related to the personal evolution of COVID-19 using a combination of survival analysis and Bayesian inference. Results shows how the disease evolved during the first year of the epidemic and how the age factor influences TVC, LoS and DH.

These results can be exploited in epidemiological model, like SIR one and its extensions in order to provide accurate estimation of the evolution of the epidemic and partially solve the non identifiability problem which often makes the predictions unstable. A little example of this approach has been proposed in case of a Social SIR model; however, this approach can easily be extended to other epidemiological parameters when the richness of datasets allows computation of specific quantities.

## Figures and Tables

**Table 1:** Descriptive statistics for TVC, LoS and DH by age factor.

(a) TVC				(b) LoS			
**Age group**	**Median**	**Q1**	**Q3**	**Age group**	**Median**	**Q1**	**Q3**
0–24	12	16	22	0–24	0	1	4
25–49	12	17	23	25–49	0	2	10
50–64	13	19	26	50–64	1	9	16
65–74	13	20	29	65–74	5	11	20
75+	11	23	40	75+	4	9	18
**(c)** **DH**							
**Age group**	**Median**	**Q1**	**Q3**				
0–24	0	0	0				
25–49	0	0	2				
50–64	0	0	4				
65–74	0	1	5				
75+	0	1	5				

**Table 2:** Descriptive statistics for TVC, LoS and DH by period.

(a) TVC				(b) LoS			
**Period**	**Median**	**Q1**	**Q3**	**Period**	**Median**	**Q1**	**Q3**
Period 1	13	25	42	Period 1	3	8	16
Period 2	16	23	35	Period 2	1	8	16
Period 3	12	17	23	Period 3	3	10	17
**(c) DH**							
**Period**	**Median**	**Q1**	**Q3**				
Period 1	0	0	1				
Period 2	0	0	2				
Period 3	0	1	7				

**Table 3:** 95% Credible Intervals for the mean of TVC.

(a) Age factor		(b) Period factor	
**Age Group**	**95% C.I.**	**Period**	**95% C.I.**
0–24	$\left[17.2,18.9\right]$	Period 1	$\left[30.8,33.9\right]$
25–49	$\left[17.8,21.3\right]$	Period 2	$\left[29.7,32.6\right]$
50–64	$\left[20.4,23.9\right]$	Period 3	$\left[23.1,24.0\right]$
65–74	$\left[21.4,24.7\right]$		
75+	$\left[28.5,32.8\right]$		

**Table 4:** 95% Credible Intervals for the mean of LoS.

(a) Age Factor		(b) Period Factor	
**Age Group**	**95% C.I.**	**Period**	**95% C.I.**
0–24	$\left[4.4,6.2\right]$	Period 1	$\left[9.8,12.4\right]$
25–49	$\left[6.0,8.7\right]$	Period 2	$\left[10.8,11.9\right]$
50–64	$\left[10.2,13.0\right]$	Period 3	$\left[10.5,12.7\right]$
65–74	$\left[12.6,16.1\right]$		
75+	$\left[11.3,13.9\right]$		

**Table 5:** 95% Credible Intervals for the mean of DH.

(a) Age Factor		(b) Period Factor	
**Age Group**	**95% C.I.**	**Period**	**95% C.I.**
0–24	$\left[0.9,1.6\right]$	Period 1	$\left[1.9,2.5\right]$
25–49	$\left[2.1,3.3\right]$	Period 2	$\left[1.7,2.4\right]$
50–64	$\left[2.4,3.9\right]$	Period 3	$\left[3.3,4.7\right]$
65–74	$\left[2.8,4.1\right]$		
75+	$\left[2.7,3.5\right]$		

## Data Availability

Data are provided by the Health Protection Agency (ATS) of Pavia.
